# Research on a Weighted Gene Co-expression Network Analysis method for mining pathogenic genes in thyroid cancer

**DOI:** 10.1371/journal.pone.0272403

**Published:** 2022-08-01

**Authors:** Bo Wang, Wei Jiang, Xiaodong Zheng, Yu Han, Runjie Liu

**Affiliations:** College of Computer and Control Engineering, Qiqihar University, Qiqihar, People’s Republic of China; University of Toronto, CANADA

## Abstract

Thyroid cancer (TC) is one of the most common thyroid malignancies occurring worldwide, and accounts for about 1% of all the malignant tumors. It is one of the fastest growing tumor and can occur at any age, but it is more common in women. It is important to find the pathogenesis and treatment targets of TC. In this pursuit, the present study was envisaged to investigate the effective carcinogenic biological macromolecules, so as to provide a better understanding of the occurrence and development of TC. The clinical and gene expression data were collected from The Cancer Genome Atlas (TCGA). We clustered mRNA and long non-coding RNA (lncRNA) into different modules by Weighted Gene Co-expression Network Analysis (WGCNA), and calculated the correlation coefficient between the genes and clinical phenotypes. Using WGCNA, we identified the module with the highest correlation coefficient. Subsequently, by using the differential genes expression analysis to screen the differential micro-RNA (miRNA), the univariate Cox proportional hazard regression was employed to screen the hub genes related to overall survival (OS), with P < 0.05 as the statistical significance threshold. Finally, we designed a hub competitive endogenous RNA(ceRNA) network of disease-associated lncRNAs, miRNAs, and mRNAs. From the results of enrichment analysis, the association of these genes could be related to the occurrence and development of TC, and these hub RNAs can be valuable prognostic markers and therapeutic targets in TC.

## Introduction

Thyroid cancer (TC) is the most common endocrine system malignant tumor, and is the endocrine tumor with the fastest growth rate in the world [1,2]. Although the overall survival rate of TC patients is better, the incidence rate is increasing significantly [3,4], and it occurs frequently in women [5]. The age standardized statistics show that, the global incidence of thyroid cancer was 2.2/100,000 men/year and 4.4/100,000 women/year. The incidence increased by 50% from 2006 to 2016, being the fastest increase among all malignant solid tumors [6]. In 2019, thyroid cancer ranked third among the female malignancies [7]. However, the overall survival rate is good, and most TCs can be effectively controlled by treatments such as surgery and endocrine suppression. Nevertheless, the mortality rate of advanced thyroid cancer still remains an important concern. Thus, it is very important to explore the potential molecular mechanisms involved in TC, so as to find effective diagnostic and therapeutic targets.

In the past few decades, the research on TC has mainly been based on molecular characteristics, morphological characteristics and immunological characteristics to explore the tumor-associated protein coding genes. However, in mammals, the protein-encoding RNA covers only 2% of the total genome. Therefore, it is necessary to discover the functions of non-coding RNA (ncRNA), including the long non-coding RNA (lncRNA) and the micro-RNA (miRNA) [8,9]. In the past, lncRNA was supposed to belong to the noise on the road of gene sequencing analysis, and the related research on ncRNA mainly focused on the miRNA. Subsequently, it was found that miRNAs could regulate the cell growth and apoptosis [10,11]. In 2005, several studies showed that miRNA were uniquely and differentially expressed in cancer tissues compared with normal adjacent tissues. For example, the expression of miRNA let-7 was down-regulated in lung cancer but not in other cancers, such as breast or colon cancer [12,13]. These studies illustrate that the expression profiling data can serve as an effective diagnostic tool for the detection of biomarkers and cancer-related genes. With the development of high-throughput sequencing technology and recent studies on lncRNAs, it has been shown that lncRNAs are closely related to the occurrence and development of various diseases and tumors [14]. Some lncRNAs were used as important regulators to participate in the whole process of life activities, and even some lncRNAs were directly regarded as biomarkers in the clinical trials [15]. For example, lncRNA PCA3, which is highly upregulated and specific to prostate cancer, was detected in the urine with levels that correspond to the severity of prostate cancer [16]. In 2011, Sal-mena et al. [17] proposed the competitive endogenous RNA (ceRNA) hypothesis, wherein specific RNAs, such as lncRNA, circular RNA (circRNA) and pseudogenes competed with the miRNA through miRNA response element (MRE), thereby affecting the gene silencing caused by miRNA. Therefore, the pathogenic mechanism of ceRNA has attracted a lot of attention. In many malignant tumors such as liver cancer and lung adenocarcinoma, the mechanism of tumor occurrence and development caused by lncRNA-miRNA-mRNA has been elucidated [18,19]. Studies have reported that a novel ceRNA activity of lncRNA MEG3 in human gastric cancer cells, lncRNA MEG3 inhibits the cell proliferation, migration and invasion in gastric cancer by competitively binding the miR-181 family, upregulating Bcl-2, and suppressing gastric carcinogenesis [20]. The study of Zhang et al. reported that a ceRNA module was mediated by has-miR-145 and the has-miR-16 in normal state, while has-miR-16 was replaced by three miRNAs including has-miR-34a,has-miR-29b and has-miR-15a in the prostate tumor state [21]. Interestingly, these three replaced miRNAs in tumor state are relevant in the tumorigenesis of prostate cancer [22–24]. In summary, these observations highlight the means of analyzing the dynamic regulation of ceRNA interactions in the exploration of the mechanism of tumorigenesis.

In the present study, TC sample and para-cancer samples were procured from The Cancer Genome Altas (TCGA) database, and were analyzed at the level of lncRNA, miRNA and mRNA. Weighted Gene Co-expression Network Analysis (WGCNA) was used to obtain the related lncRNAs and mRNAs with significant positive correlation to TC cancer. The significantly different miRNAs were screened out by the differential genes expression analysis. Then, the univariate Cox proportional hazard regression was employed to screen the hub mRNAs related to overall survival. Finally, a novel lncRNA-miRNA-mRNA ceRNA network was constructed for TC. These hub RNAs can be valuable prognostic markers and therapeutic targets in TC. This study comprehensively analysed the TC-related ceRNA network to allow a more convenient tool to explore the post-transcriptional regulatory mechanism underlying in TC and suggest novel biomarkers for the diagnosis, treatment, and prognosis of TC.

## Materials and methods

### Data source

The TC RNA-seq expression profile of counts type and miRNA-seq of BCGSC miRNA profile were downloaded from TCGA (Project ID: TCGA-THCA, Data Release: 29.0, https://portal.gdc.cancer.gov/). Among them, the RNA-seq consisted of 560 samples, including 58 normal samples and 502 tumor samples, and the miRNA-seq consisted of 573 samples, including 57 normal samples and 516 tumor samples. At the same time, the clinical data of 506 patients with TC was downloaded. Since RNA-seq from TCGA contains lncRNA and mRNA, the annotation files were downloaded from Gencode (Data Release: 37, https://www.gencodegenes.org/) to annotate the RNA-seq and classify lncRNA and mRNA.

### Dataset preprocessing

Initially, the RNA-seq data was annotated and classified through the annotation file downloaded by Gencode; the data consisted of 19563 mRNA and 15096 lncRNA. Because the mutated gene is usually considered as background noise, in order to reduce the impact of a small number of outliers on the overall data, we selected 5000 genes according to average expression >1 and the greatest median absolute deviation (MAD). Subsequently, we used the R language to draw a sample cluster map to see if there were any outliers and removed them. Finally, log(x+1) normalization was performed on the gene expression profiles, so that the up-regulated or down-regulated genes were continuously distributed around 0.

### Weighted Genes Co-expressing Network Analysis

Weighted Gene Co-expression Network Analysis (WGCNA) refers to a biology method used to describe the gene association patterns between different samples [25]. It is an algorithm based on high-throughput gene expression profiles. It is based on the connectivity of genes and the correlation between genes to cluster gene modules, analyzes the co-expression relationship between genes, and screens out the core genes closely associated with other gene [26]. Genes in the same module are considered to have high correlation, consistent expression level, and similar biological functions, which makes it helpful to explore the biological functions of the different modules [27]. In undirected network, the genes in the module are highly related, and in directed network, they are highly positively correlated. The undirected network was employed in this study.

We used the WGCNA- R package to calculate the independence and average connectivity of the different modules under different power values, and selected the value when the independence coefficient R^2^ reached 0.8 for the first time. In this study, the power value for mRNA modules was screened out as 7, and the power value for lncRNA modules was screened out as 6. According to the internal connectivity and soft threshold of genes, mRNA and lncRNA were clustered into different co-expression modules. Further, we calculated the correlation between the module and the clinical phenotype, and selected the module with the greatest correlation coefficient.

### Preliminary screening of related miRNAs

The redundant information of the miRNA data was removed, and only the gene expression data in the sample was retained and divided into two groups according to the clinical dominance of the sample, cancer group and normal group. Using “Limma” R package, we performed the differential genes expression analysis of miRNA expression profile, identified the genes that were differentially expressed in normal and cancer tissues. 79 miRNA were screened out exhibiting a significant correlation with TC at P<0.05.

### Usage of databases

By WGCNA, we obtained the mRNA module and lncRNA module that exhibit a highly significant correlation. miRcode [28] was used to predict the targeted miRNA by lncRNA in the lncRNA module with significant correlations.Targetscan [29] and miRDB [30] were used to predict mRNAs associated with miRNA.

## Results

### Clustering of mRNA co-expressing module

**Fig 1:**
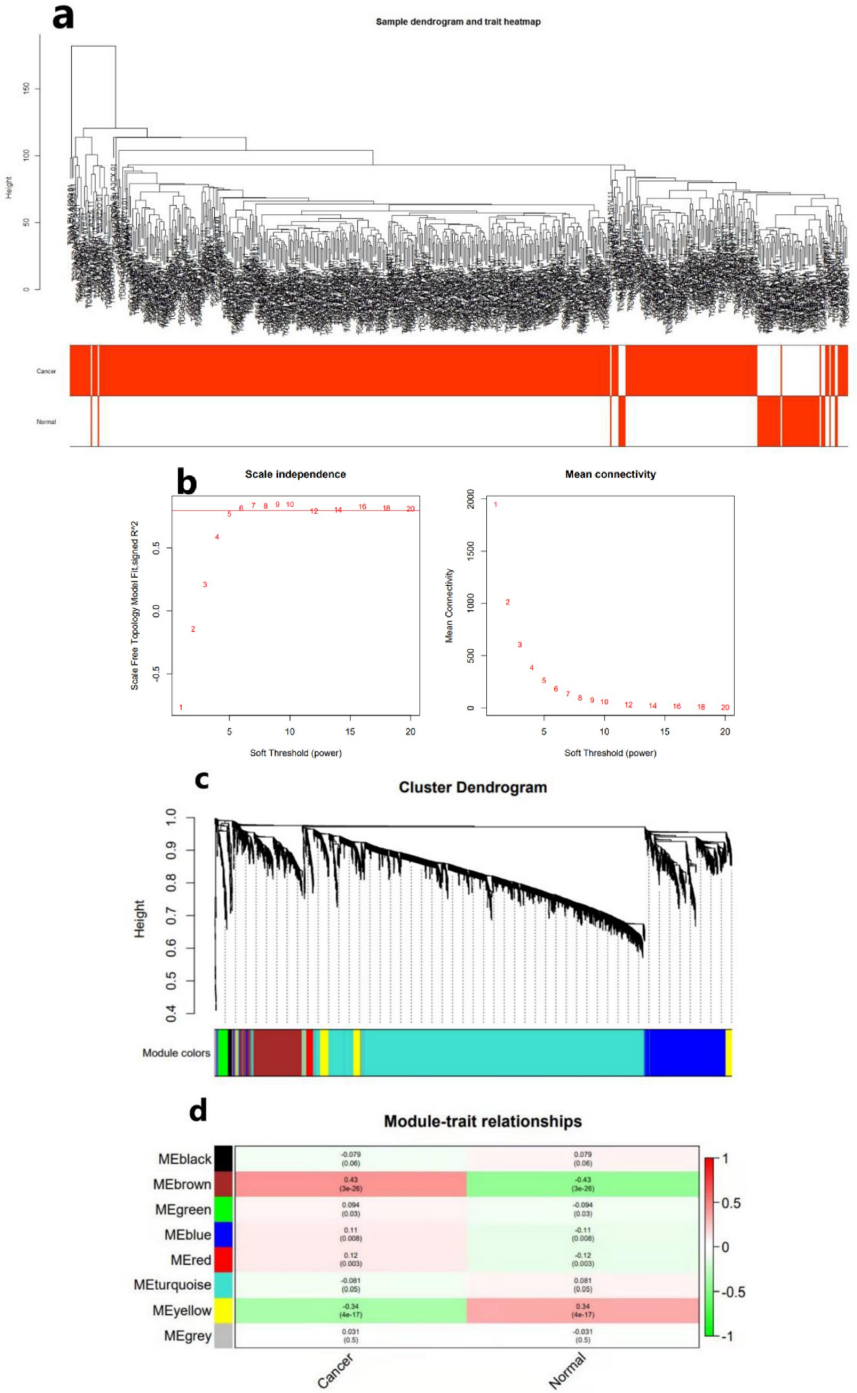
The process of screening TC-related mRNAs using WGCNA. (a) Hierarchical clustering for TC samples constructed based on their mRNA expression levels. (b) Selecting for soft threshold(power). When the power value is 7, the degree of independence was > 0.8 for the first time. (c) mRNA co-expression modules clustering and dividing by using WGCNA. (d) Correlation analysis between modules and clinical phenotypes.

From the mRNA expressing profiles of 560 TC cases in TGCA database, 19563 mRNA were selected according to MAD within the top 5000 with an average expression > 1. The samples that were already metastasized were removed in the construction of co-expression modules using WGCNA. First, we performed the sample cluster analysis [31] on the data to see if there were outliers; the sample clustering is shown in Fig 1A. Next, we selected the power value which affects the independence and average connectivity, according to the result of Fig 1B and selected the power value that was suitable for the undirected networks. After testing, it was found that when the power value was 7, the degree of independence was > 0.8 for the first time and it best met the requirements of the scale-free network. We used the power value to construct a weighted gene co-expression network. Eight modules were divided by this network as shown by Fig 1C. The correlation analysis between the downloaded clinical data of TC from TCGA and the constructed modules showed that the brown module exhibited a significant correlation with the clinical phenotype of TC; the correlation coefficient is shown in Fig 1D. The brown module consisted of 573 genes (WGCNA-mRNA). As a result, it was initially believed that 573 mRNAs exhibited a significant correlation with the TC samples.

### Clustering of LncRNA co-expressing module

**Fig 2:**
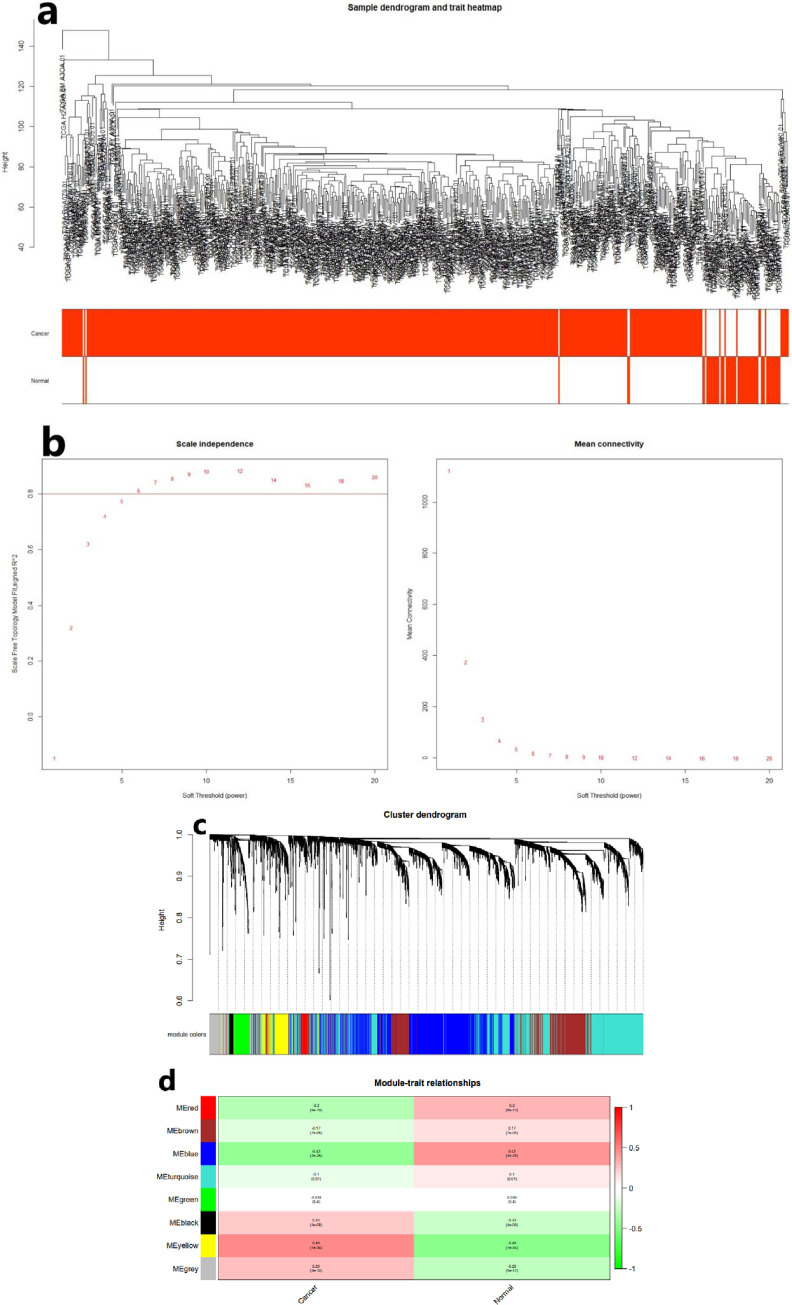
The process of screening TC-related mRNAs using WGCNA. (a) Hierarchical clustering for TC samples constructed based on their lncRNA expression levels. (b) Selecting for soft threshold(power). When the power value is 6, the degree of independence was > 0.8 for the first time. (c) lncRNA co-expression modules clustering and dividing by using WGCNA. (d) Correlation analysis between modules and clinical phenotypes.

LncRNA co-expressing modules was constructed using the same method of the mRNA co-expressing module. After pre-processing the lncRNA expression profiles, we clustered them hierarchically and observed the sample distribution; the cluster tree is shown in as Fig 2A. Subsequently, we chose a power value suitable for lncRNA expression profiles to construct a co-expression network. As shown in Fig 2B, the power value of 6 was found to be the most appropriate. Using this power value to construct a co-expressing network, and dividing the genes into modules, a total of 8 modules were obtained, as shown in Fig 2C. It can be seen from Fig 2D, the yellow module exhibited a highest positive correlation coefficient with TC, and the module contains 220 genes (WGCNA-lncRNA).

### Preliminary screening of miRNA

**Fig 3:**
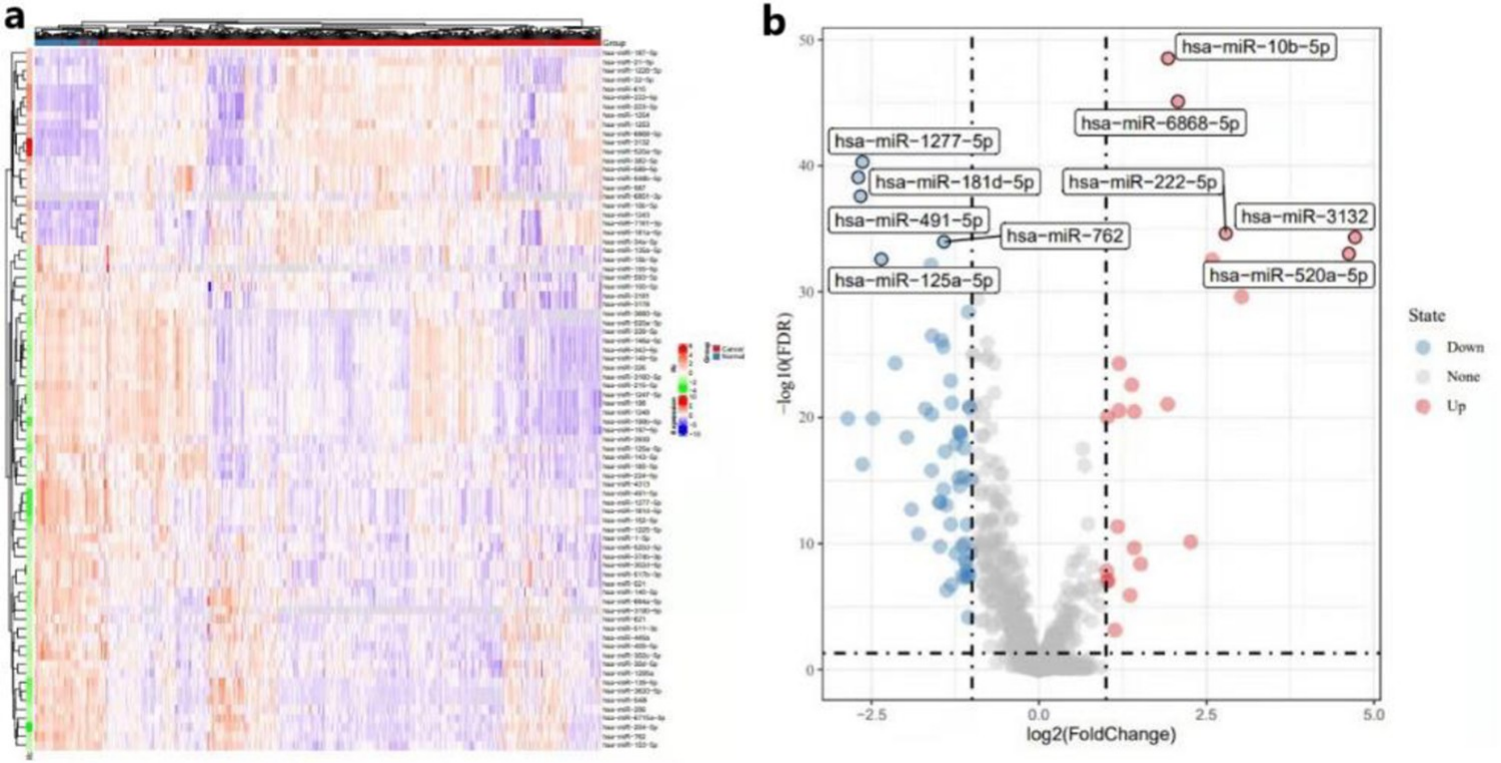
The differential genes expression analysis on miRNA. (a) The overall expression level of miRNA. (b) The volcano diagram about the result of the differential genes expression analysis on miRNA.

Because gene mutations become the biggest noise in the analysis of cancer-causing genes, we removed the genes whose expression level was 0 in 75% of the samples, and log(x+1) normalized the data. Subsequently, we constructed a cluster heat map as shown in Fig 3A to observe the overall expression level of miRNAs. Finally, we performed the differential genes expression analysis on the pre-processed miRNA, and obtained 78 differential genes (DEG-miRNA); the volcano plot of DEG-miRNAs is shown in Fig 3B.

### Prediction of target relationship base on network databases

Considering the large scale of the data, the database files were downloaded to the local for manual comparison, and we used the loop statement to screen them one by one, so as to obtain the common genes.

**Fig 4:**
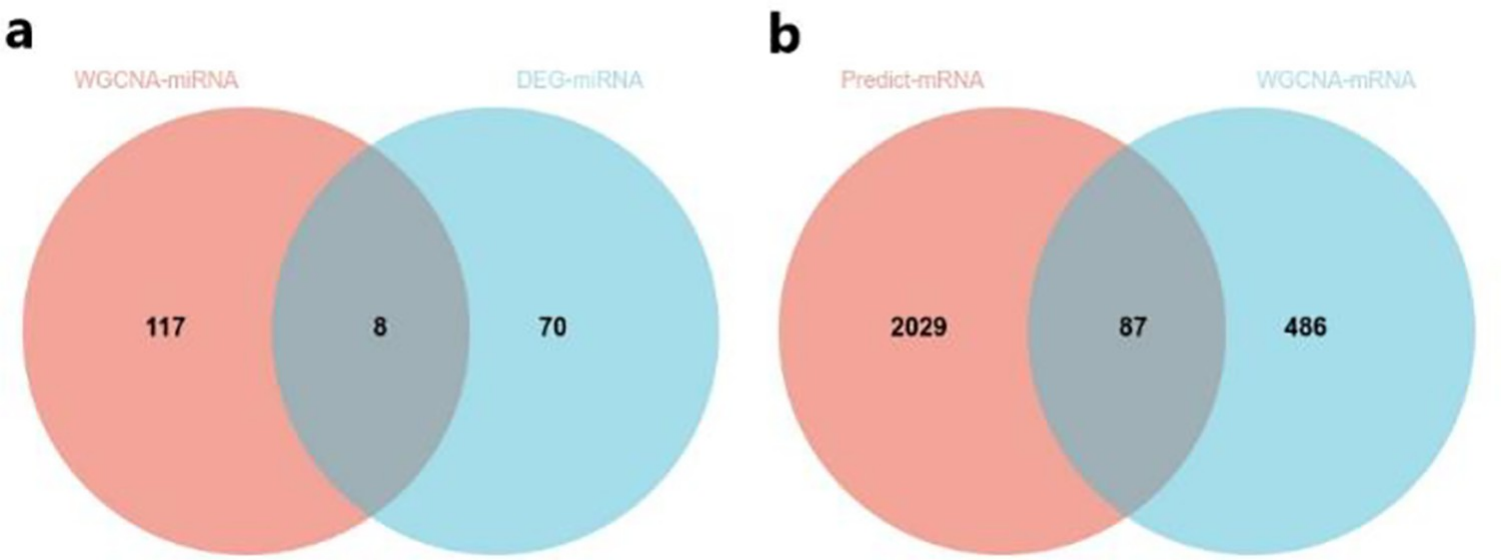
Wayne diagram of gene comparison result. (a) Wayne diagram was employed to intersect WGCNA-miRNAs and DEG-miRNAs and obtained 8 common miRNAs. (b) Wayne diagram was employed to intersect Predict-mRNAs and WGCNA-mRNAs and obtained 87 common mRNAs.

miRcode was used to predict the miRNA target of 220 WGCNA-lncRNAs, and we obtained 125 miRNA targets (Predict-miRNAs) that exhibited a binding with them. We then compared them with 78 DEG-miRNAs obtained from the differential genes expression analysis to get the intersection, and constructed the Wayne diagram as shown in Fig 4A. From the figure, we got 8 miRNA.

TargetScan and mirDB were used to predict the target mRNA of miRNA. In order to better confirm the accuracy of the prediction results, we selected the mRNA from the intersection of the two database predictions, and 2116 mRNA were obtained. Similarly, we compared these 2116 mRNAs with 573 WGCNA-mRNAs, and as shown in the Fig 4B, we obtained 87 mRNA.

### Univariate cox regression screening for hub-mRNAs

Univariate Cox proportional hazard regression model was used to screen out the preliminary related-mRNAs by verifing the correlation between the expression levels of these 87 common mRNAs and the overall survival (OS). The threshold P value was set to 0.05.

**Fig 5:**
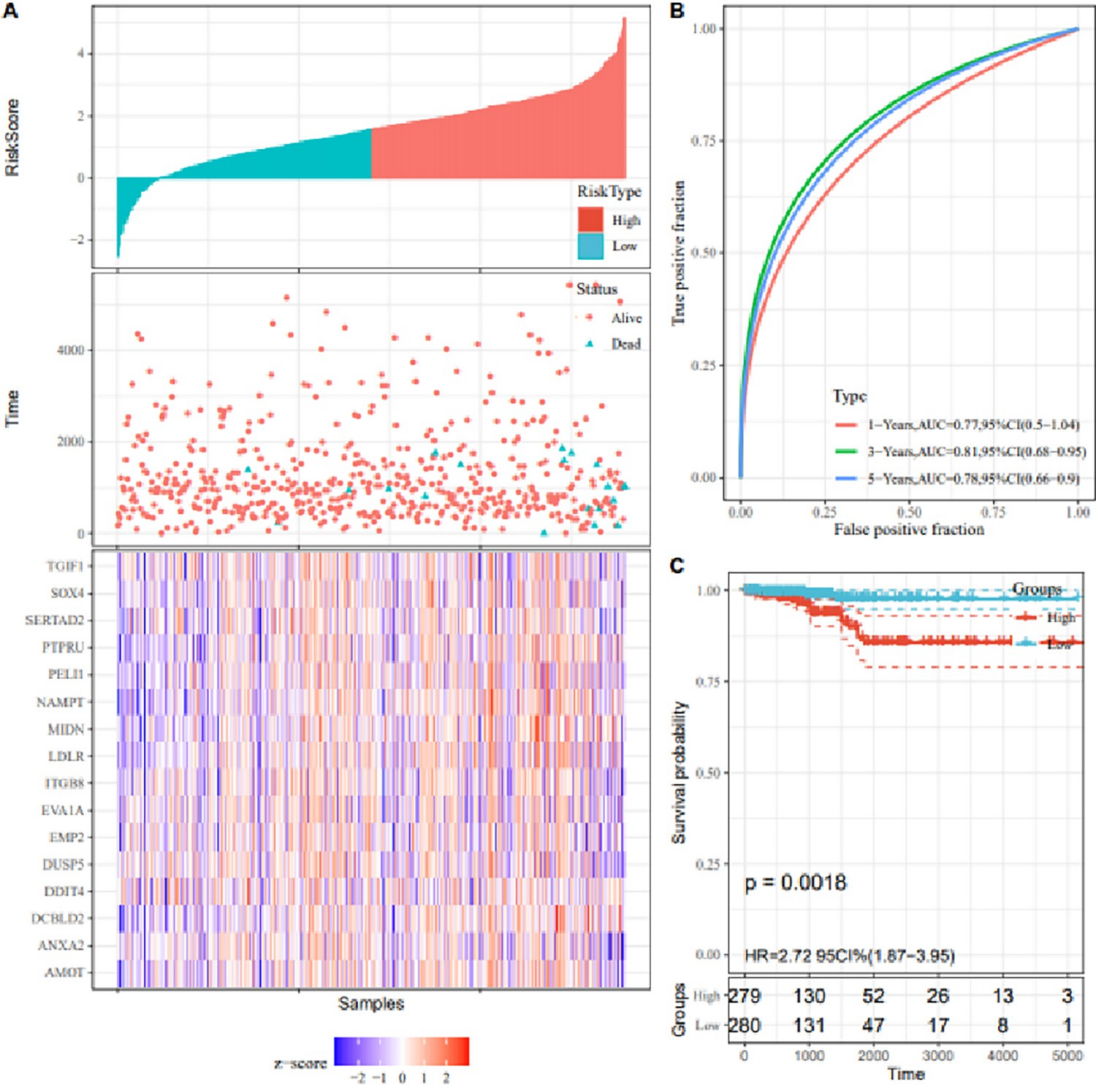
Survival prediction model for screening hub-mRNAs. (a)The scatter plots of overall sample distribution, risk score plots, and gene expression heatmaps for high-risk and low-risk categories.The majority of death samples have high risk factors (b)The ROC curves based univariate Cox regression. (c) Survival curves of the high-risk and low-risked.

The R package “survival” was used to construct the univariate Cox proportional hazard regression model.As shown in Table 1, 17 genes with P value <0.05 were obtained from the univariate Cox proportional hazard regression model. Then, we divided the TC samples into high-risk and low-risk group and observed the overall distribution of the TC samples as shown in Fig 5A. To test the prediction accuracy of the model, we constructed the ROC curves (showed in Fig 5B). The survival rate of the high-risk group was significantly lower than that of the low-risk group (p = 0.0018), as shown in Fig 5C.

**Table 1:** Result of univariate survival analysis.

Gene	P-value	HR	Low 95%	High 95%
MIDN	0.00001	0.78672	0.70701	0.87543
ITGB8	0.00003	0.90470	0.86251	0.94896
LDLR	0.00055	0.91140	0.86467	0.96067
EMP2	0.00172	0.85162	0.77025	0.94158
DDIT4	0.00361	1.12602	1.03949	1.21975
TGIF1	0.00861	0.85963	0.76790	0.96232
SERTAD2	0.01172	0.85052	0.74991	0.96464
SOX4	0.01190	0.90741	0.84124	0.97879
DUSP5	0.01566	0.94427	0.90137	0.98922
PTPRU	0.01678	0.92332	0.86489	0.98571
NAMPT	0.02759	0.90748	0.83239	0.98934
EVA1A	0.02811	0.95197	0.91104	0.99473
DCBLD2	0.02895	0.88559	0.79412	0.98760
ENTPD1	0.04034	1.07242	1.00308	1.14657
ANXA2	0.04329	0.92001	0.84855	0.99748
AMOT	0.04545	0.92641	0.85957	0.99845
PELI1	0.04588	0.94069	0.88588	0.99888

### Construction of ceRNA network

**Fig 6:**
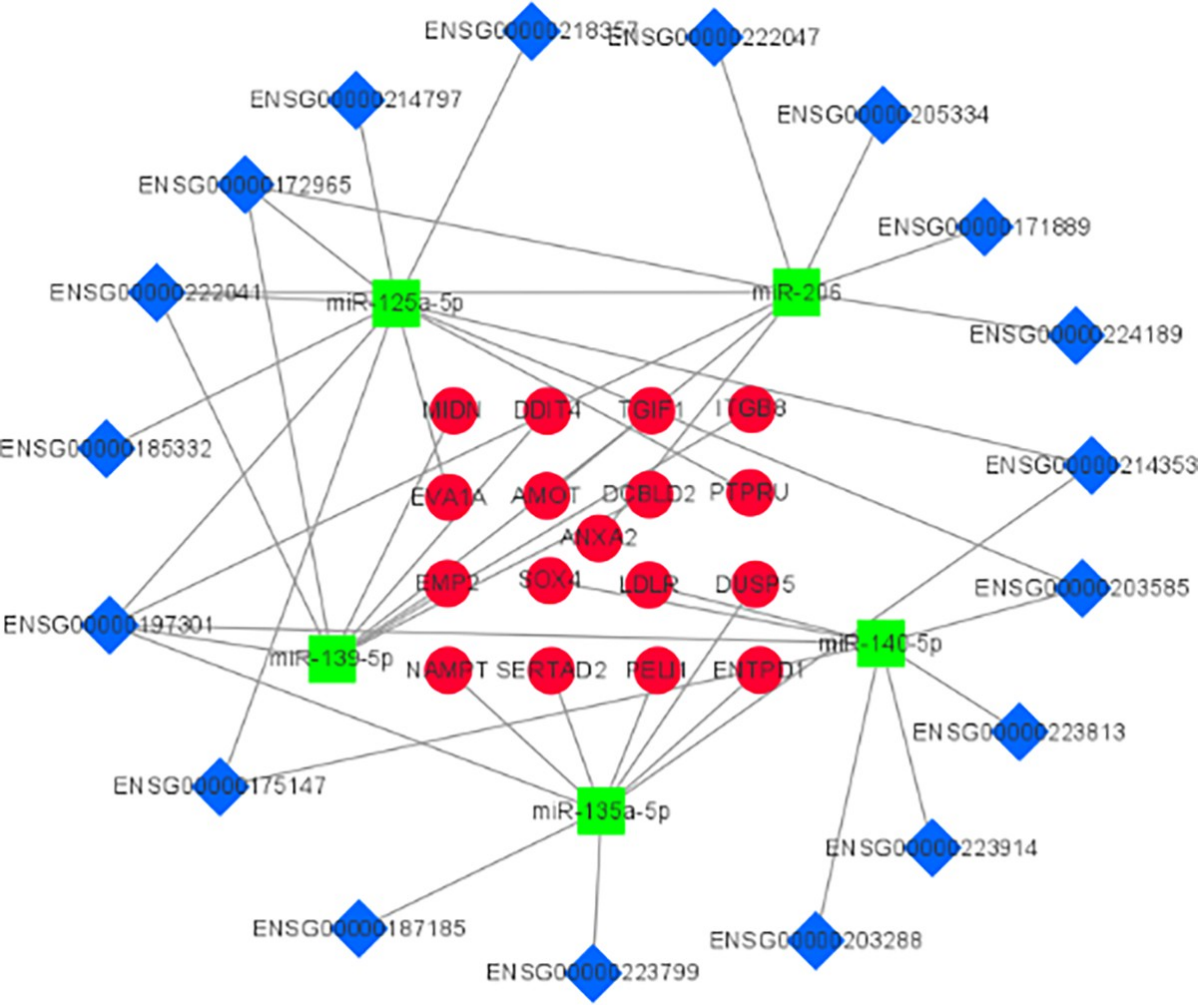
The interaction diagram of the ceRNA network.

From the comparison of the data from the experimental and database, we obtained 17 mRNAs, 19 lncRNAs and 5 miRNAs. In order to make the network structure more intuitive, the ceRNA network was performed and visualized as shown in Fig 6 using Cytoscape v3.8.2 software.

### KEGG pathway enrichment analysis

In order to further reveal the potential biological functions of mRNAs in TC, the KEGG Pathway enrichment analysis was performed on the17 mRNAs. For gene functional enrichment analysis, KEGG rest API (https://www.kegg.jp/kegg/rest/keggapi.html) was used to get the latest gene annotation, and used as a background to map the genes to the background set. Finally, the R package clusterProfiler (version 3.14.3) was employed in the enrichment analysis to obtain the result of genes enrichment [32].

**Fig 7:**
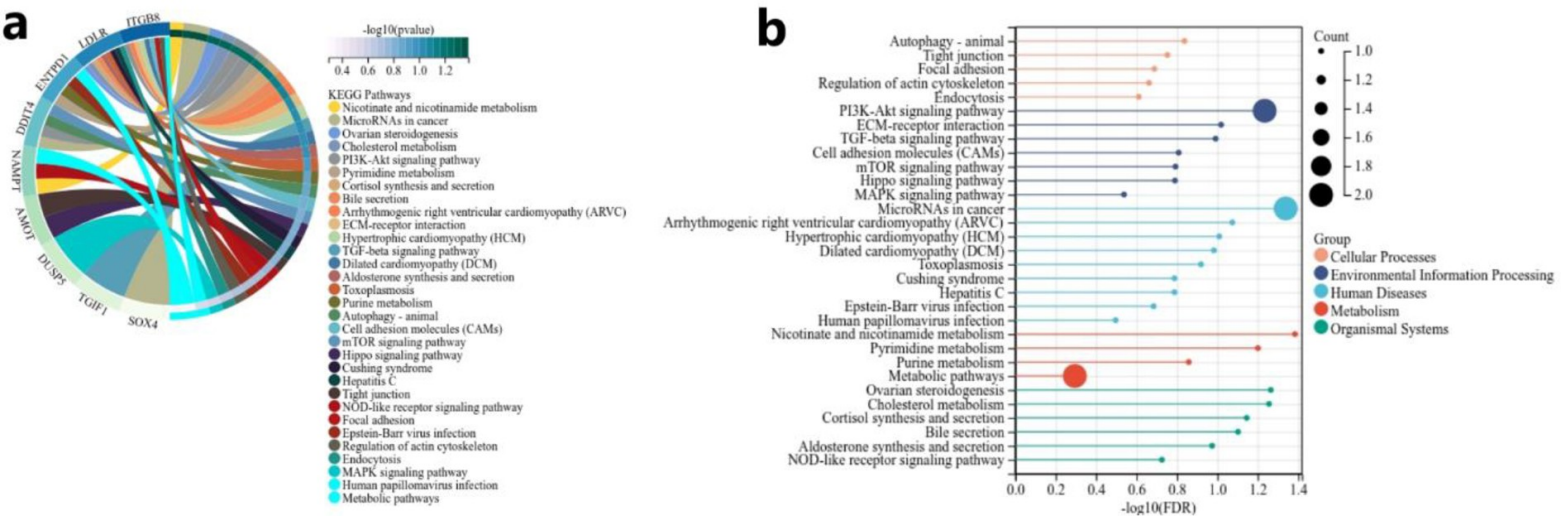
Result of KEGG pathway enrichment analysis. (a)a total of 31 KEGG pathway were enriched by 9 mRNA. (b)Bar chart of the result.

Fig 7A and 7B show that a total of 31 KEGG pathways were enriched with 9 mRNA, including some tumor-related, such as PI3K-Akt signaling pathway, microRNAs in cancer, mTOR signling pathway and so on.

### Survival analysis

**Fig 8:**
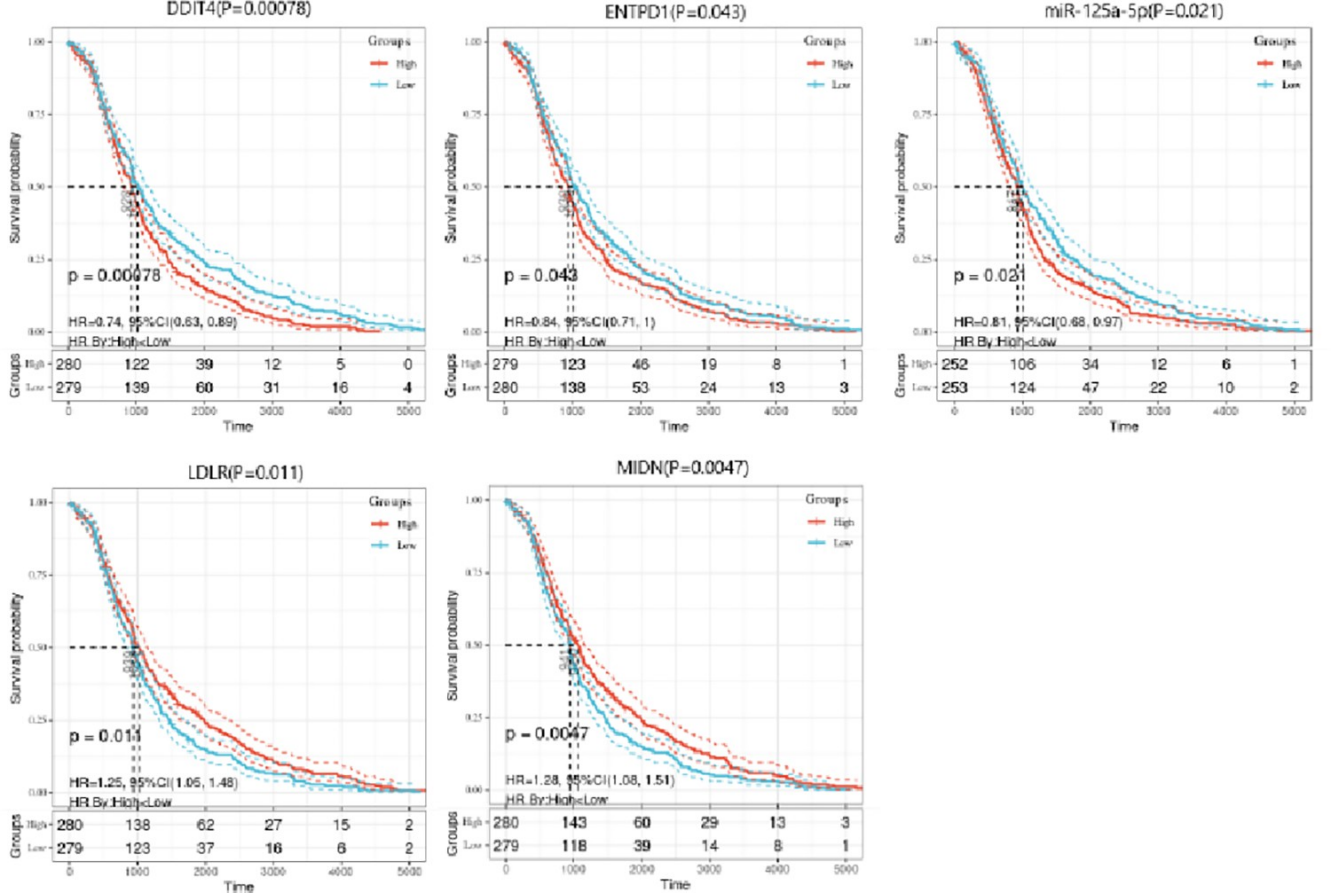
Survival curve of 4 mRNA and 1 miRNA.

In the present study,the R package “Survival” was employed, and we integrated the survival time, survival status and gene expression data in the dataset. The prognostic significance of all mRNA, miRNA in the ceRNA network and overall survival was evaluated using P < 0.05 as the cut-off threshold. Fig 8 reveals the Kaplan-Meier survival curves, which show that 4 mRNAs (DDIT4, ENTPD1, LDLR, MIDN) and 1 miRNA (miR-125a-5p) exhibited a significant impact on the prognosis of TC patients.

**Fig 9:**
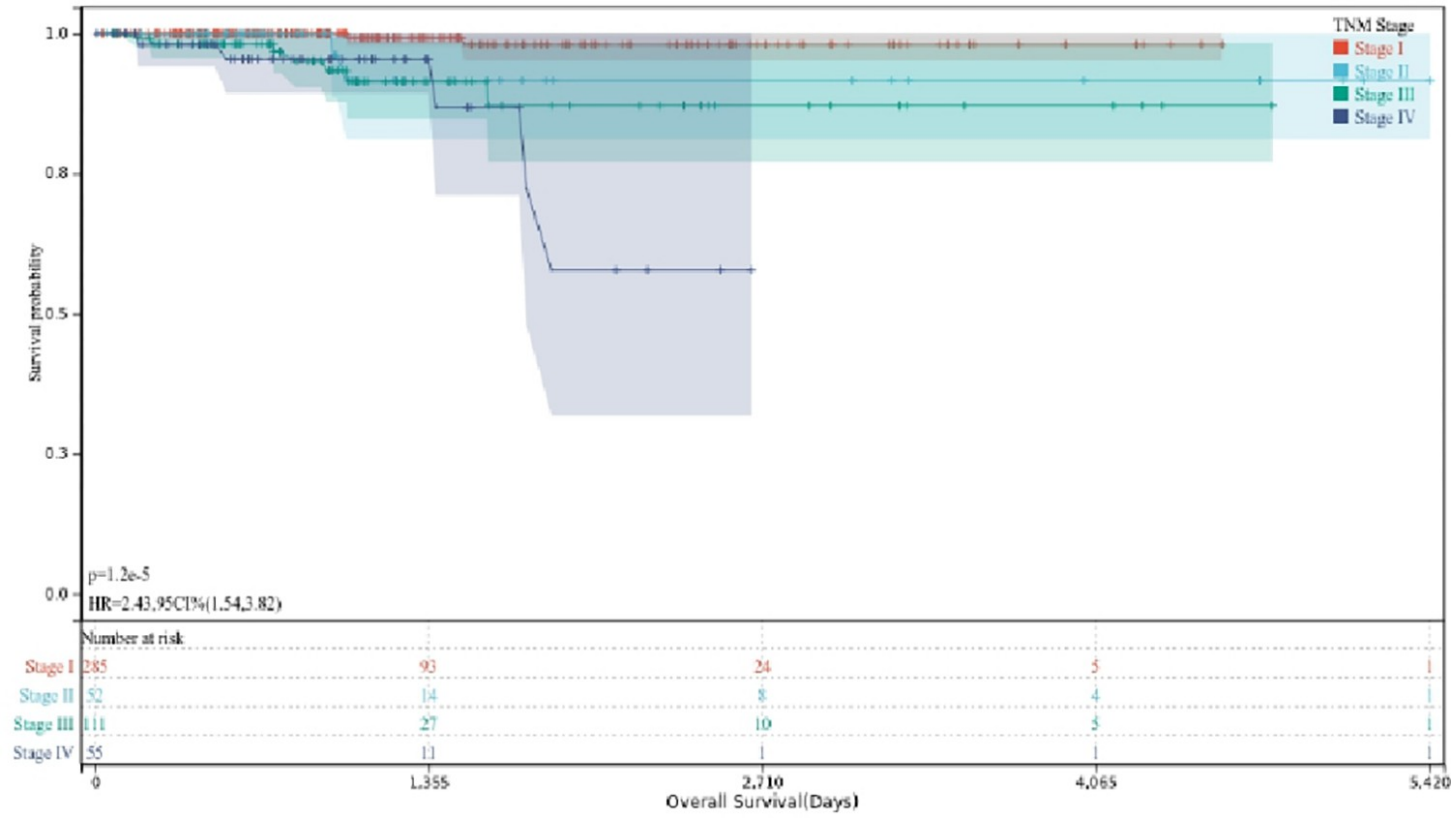
Survival curve of TNM stage.

**Fig 10:**
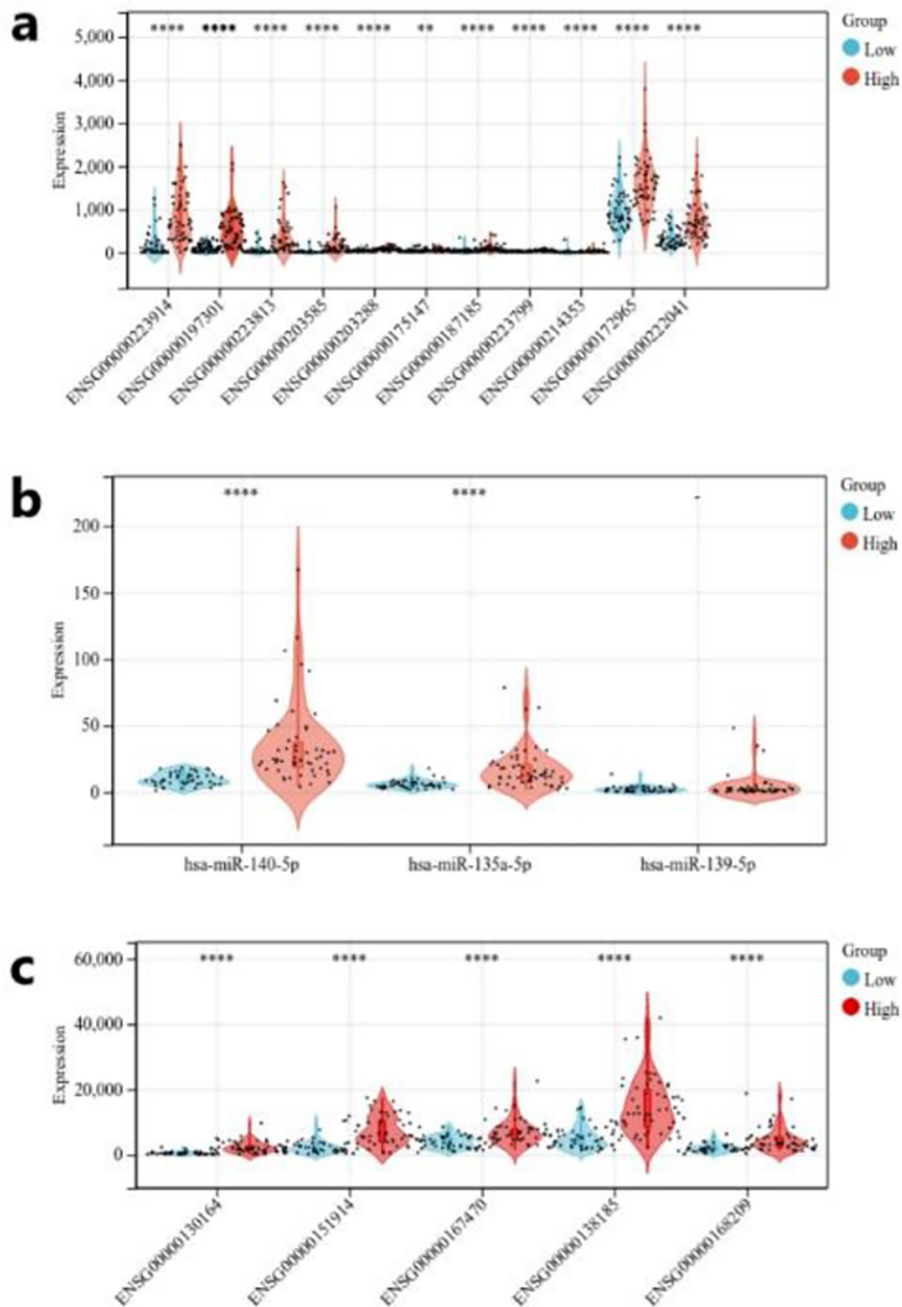
Comparison of genes in ceRNA network in different TNM stages. The number of asterisks represents the degree of difference (a)Differential expression of lncRNAs contained in the ceRNA network in low-risk samples and high-risk samples(b) Differential expression of miRNAs contained in the ceRNA network in low-risk samples and high-risk samples(c) Differential expression of mRNAs contained in the ceRNA network in low-risk samples and high-risk samples.

Considering that the prognosis of different types of thyroid cancer is quite different, all cancer samples were divided into four parts based on TNM stage (2017 AJCC, 8^th^ ed.). After sorting out, we found that stage I, II and III belonged to the differentiated stage, and stage IV belonged to the anaplastic stage. Survival analysis was performed in four parts. The results are shown in the Fig 9, and it can be seen that the survival probability of stage IV was significantly lower than stage I, II, III. From these results, it is clear that the anaplastic thyroid cancer has a worse prognosis than the differentiated thyroid cancer. Based on the above results, we defined stage IV as the high-risk group and stage I, II and III as the low-risk group. Subsequently, we made two groups of cancer samples of equal size with high risk and low risk, and consteructed the violin and scatter plots combined with gene expression data; the results are shown in Fig 10. Onobserving the overall expression levels of the ceRNA internal genes in the two groups of samples, it can be found that most of the key genes in the high-risk group were much higher than those in the low-risk group. To a certain extent, this reflects the role of key genes in the different stages of TNM, that is to say, the overexpression of key genes greatly reduced the overall survival rate of patients.

**Fig 11:**
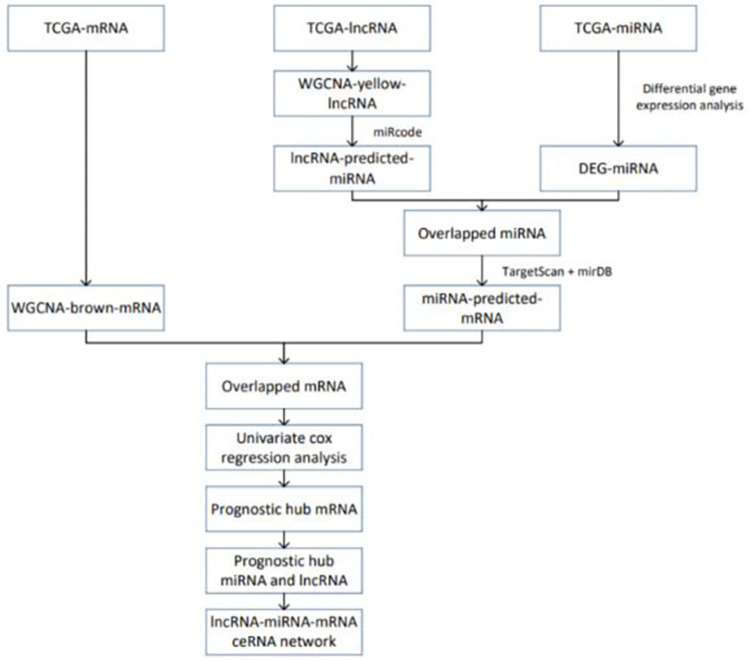
The analysis flow of the study.

The overall flow of the experiment is shown in the Fig 11.

## Discussion

Thyroid cancer (TC), as the most common endocrine system malignant tumor, is mainly prevalent in the children and teenagers [4]. The incidence in women is about 3 times that of the incidence in men [33]. Although the death rate of TC is lower than many other cancers, the death rate in the terminal stage of TC is not optimistic. Therefore, it is very essential to study and find the potential pathogenic mechanisms and therapeutic targets of TC.

At present, the main method of clinical research on malignant tumors is gene sequencing, wherein the tumor samples are sequenced to determine whether there are any genetic mutations and accordingly confirm the target treatment plan. Numerous recent studies have proved that lncRNA can competitivelly bind the miRNA to regulate the expression of the target genes based on the ceRNA hypothesis [34,35]. These confirmations provide a reference for exploreing the mechanism of lncRNA, miRNA and mRNA in TC. In the past, ther research on TC was mainly focused on mRNA, and there were few studies on lncRNA [36]. Although a few studies have shown that there are some genes that are significantly differentially expressed in TC, most of them have not shown the mechanism of these genes in the process of the tumor development. This phenomenon has been alleviated with the development of high-throughput sequencing technology, but correspondingly, the development of high-throughput sequencing technology generates a large amount of sequencing data, which ist difficult for subsequent analysis and interpretation of biological problems.

Recent studies have shown that the ceRNA network in TC plays a regulatory role and corresponding mechanisms in the development of cancer, such as lncRNA MALAT1 function as a ceRNA via sponging miR-200a-3p to derepress the FOXA1 expression [37]. Further, linc01278 is known to sponge miR-376c-3p to positively regulate the DNM3 expression and ultimately acting as a tumor suppressor gene in papillary thyroid carcinoma [38]. SNHG3 binds to the miR-214-3p target in regulating the proteasome 26S subunit non-ATPase expression [39]. These studies have proved that there are pathogenic mechanisms rellated to the regulation of ceRNA in TC. However, there is a need of additional studies to systematically explore the internal regulatory mechanism of the ceRNA.

In this study, WGCNA was used to screen the modules of mRNA and lncRNA that showed a significant trend in the development of TC, including one brown module of mRNA and one yellow module of lncRNA. At the same time, the differential genes expression analysis was used to screen the different miRNAs. Using the interactions of lncRNA-miRNA-mRNA, we constructed a ceRNA network, and then we analyzed it by KEGG enrichment and survival analysis.

From the KEGG pathway enrichment analysis, we found that 17 mRNAs in the ceRNA network were mainly enriched in 31 pathways. Among these pathways, lipid metabolism is one of the TC characteristics [40]. In addition, many molecular signaling pathways of these pathways play an important role in the development of tumors, such as PI3K-Akt signaling pathway and the mTOR signaling pathway [25]. Many other pathways are enriched in different cancer pathways and miRNA pathways related to cancer. Thus, these genes may promote the development of TC through ceRNA.

According to relevant literature, miRNAs in the ceRNA network is involved in the regulation of cancer occurrence and development, such as miR-206 can inhibit the proliferation and invasion of thyroid cancer by targeting RAP1B [41]. miR-139-5p not only plays an important part in tumorigensis processes, but also serves as a biomarker with sufficient sensitivity and specificity in the diagnosis of cancer in clinical setting [42]. Zheng et al. found that lncRNA DANCR plays an oncogenic role in the progression of tongue squamous cell carcinomavia targeting miR-135-5p [43]. miR-140-5p suppresses the tumor growth and metastasis by inhibiting the transforming growth factor beta (TGF-β) and mitogen-activated protein kinase/extracellular signal-regulated kinase (MAPK/ERK) signaling [44]. Further, the miR-125a-5p levels in breast cancer can be a useful prognostic biomarker and offer a novel therapeutic avenue by targeting the HDAC4 in breast cancer [45].

In this study, the Kaplan-Meier survival curves showed that 4 mRNA and 1 miRNA exhibited a noteworthy impact in the prognosis of the patients. Among these genes, DDIT4 activates the mTOR signaling pathway to promete the glioma progression [46]. ENTPD1 serves as a metabolism-related gene and plays a critical role in TC through regulating metabolic pathways [47]. Mutations in the LDLR gene can cause abnormal lipoprotein metabolism, and the expression imbalance of LDLR may be related to the development of cancer [48]. MIDN promotes the expressing of parking E3 ubiquitin ligase, and MIDN loss can trigger Parkinson’s disease [49]. In addition, from to the database comparison process and ceRNA network visualization process, we can know all the genes that are related to the corresponding miRNA. The above studies indicate that these genes may be used as potential biomarkers of TC, which can be beneficial in the diagnosis and treatment of TC patients.

The aim of the present study was to find the genes closely related to the occurrence and development of TC. However, considering that most of the genes and diseases are not directly related, we conducted our study based from the perspective of ceRNA network and then elucidated the mechanism by which these genes interact to cause cancer. At the same time, we combined the clinical data to study the influence of these genes on the prognosis of TC patients to make the study more meaningful in a clinical setting. Although this study highlights the prognostic value of this hub ceRNA network, there are certain limitations of this study. First, as the sample data was limited, some genes in this ceRNA network were found to be regulators in other cancers. However, there was no significant difference in the TC patient survival, when a few genes overexpressed or underexpressed in the TC samples. At the same time, our research was limited considering the relationship between the known genes. In order to solve these problems, computing models can play a vital role, which needs a deeper study. Many studies have used known interactions between the genes to predict human disease-related ncRNAs. For example, Chen et al. [50] used the known miRNA similarity and disease similarity to construct the miRNA-disease associations, in order to predict the disease-related miRNAs. Follow-up studies have focused on lncRNA-miRNA interactions, such as Zhang et al. [51] applied a semi supervised interactome network-based approach to explore and forecast the latent interaction between lncRNAs and miRNAs. Zhang et al. [52] developed a network distance analysis model for the lncRNA-miRNA association prediction (NDALMA). Similarity networks for lncRNAs and miRNAs were calculated and integrated using the Gaussian interaction profile (GIP) kernel similarity. Similarly, Liu et al. [53] established a novel matrix factorization model to predict the lncRNA-miRNA interactions, namely the lncRNA-miRNA interactions prediction by logistic matrix factorization with neighborhood regularized (LMFNRLMI). Wang et al. [54] conducted analysis based on circRNA, and used the known associations of various diseases to build computational models to predict more potential associations. And some studies [55] are to improve the existing algorithms to make them perform better in predicting gene-disease association. The advantage of these studies is that they were not limited to a single disease and individual type gene, and a large amount of useful information can be mined, with high reliability and stability. In future studies, we can combine the known gene-disease association, as well as the gene-gene association, and use multivariate datasets to build computational models to predict whether there is an association between the lncRNA-miRNA-mRNA gene pairs and diseases. The purpose of this study was to committed to the development of medicine, we first assume that all the samples there is no abnormal clinical data, but still exist in the process of data processing the phenomenon of lack of some clinical data, we eliminate the abnormal data, at the same time we analyze the factors considered in the process of too little, the subsequent will consider into more clinical factors [56].

## Conclusions

The present study established the lncRNA-miRNA-mRNA network in TC to explore the pathogenic mechanism of ceRNA that may be related in the development of TC. The results showed that the genes contained in the ceRNA network were significantly associated with patients’ survival and TNM staging, which could help doctors make clinical treatment decisions for patients. Meanwhile, our research process could be evaluated at a lower cost than conventional gene screening methods such as gene sequencing.

The ceRNA network was established by multiple screening models, yielding network components with high prognostic value for TC. More importantly, it comprehensively elaborates the post-transcriptional regulatory mechanisms involved in TC and the ceRNA network proposed in this study can provide a reference and direction in the clinical setting, and provides potential biomarkers in the diagnosis, treatment and prognosis of TC patients.

## Supporting information

S1 Dataset (ZIP)

## References

[1] Tavares C, Eloy C, Melo M, et al., “mTOR pathway in papillary thyroid carcinoma: Different contributions of mTORC1 and mTORC2 complexes for tumor behavior and SLC5A5 mRNA expression,” *Int J Mol Sci*, vol. 19, no. 5, pp. 1448–1448, 2018. 10.3390/ijms19051448 PMC5983778 29757257

[2] Wen D., Liao T., Ma B., et al., “Downregulation of CSN6 attenuates papillary thyroid carcinoma progression by reducing Wnt/β‐catenin signaling and sensitizes cancer cells to FH535 therapy,” *Cancer Medicine*, vol. 7, no. 2, pp. 285–296, 2018. doi: 10.1002/cam4.1272 29341469 PMC5806103

[3] Fagin JA and Wells SA, “Biologic and Clinical Perspectives on Thyroid Cancer,” *New England Journal of Medicine*, vol. 375, no. 23, pp. 1054–1067, 2016. 27626519 10.1056/NEJMra1501993 PMC5512163

[4] Siegel R. L. and Miller K. D. and Jemal A., “Cancer statistics,” *CA Cancer J Clin*, vol. 67, no. 1, pp. 7–30, 2020 10.3322/caac.21387 28055103

[5] Malaguarnera R., Ledda C., Filippello A., et al., “Thyroid cancer and Circadian Clock Disruption,” *Cancers*, vol. 12, no. 11: 3109, 2020. 10.3390/cancers12113109 PMC7690860 33114365

[6] Davies L. and Welch H. G., “Current thyroid cancer trends in the United States,” *JAMA Otolaryngol Head Neck Surg*, vol. 140, no. 4, pp.317–322, 2014. doi: 10.1001/jamaoto.2014.1 24557566

[7] Peng X., Zhang K., Ma L., et al., “The role of long noncoding RNAs in thyroid cancer,” *Front Oncol*, vol. 10: 941, 2020. 10.3389/fonc.2020.00941 PMC7300266 32596158

[8] Chen Xing, Chenggang Clarence Yan, et al. “Long non-coding RNAs and complex diseases: from experimental results to computational models.” *Briefings in Bioinformatics*, 2017, vol.18, no.4, pp.558–576, 2017. doi: 10.1093/bib/bbw060 27345524 PMC5862301

[9] Chen Xing, Xie Di, Zhao Qi and You Zhu-Hong, “MicroRNAs and complex diseases: from experimental results to computational models.” *Briefings in Bioinformatics*, 2019, 20(2):515–539. doi: 10.1093/bib/bbx130 29045685

[10] Shelton J, Cheng AM, Byrom MW, et al. Antisense inhibition of human miRNAs and indications for an involvement of miRNA in cell growth and apoptosis. *Nucleic Acids Res*, 2005, 33 (4): 1290–1297. doi: 10.1093/nar/gki200 15741182 PMC552951

[11] Tanno B, Cesi V, Vitali R, et al. Silencing of endogenous IGFBP-5 by microRNA interference affects proliferation, apoptosis and differentiation of neuroblastoma cells. Cell Death & *Differentiation*, 12(3): 213–223, 2004. 10.1038/sj.cdd.4401546 15618969

[12] Eder M., Scherr M. “MicroRNA and lung cancer.” *N*. *Engl*. *J*. *Med*, 352(23):2446–2448, 2005. doi: 10.1056/NEJMcibr051201 15944431

[13] Johnson S.M., Grosshans H., et al “RAS is regulated by the let-7 microRNA family.” *Cell*, vol. 120, no. 5, pp. 635–647, 2005. doi: 10.1016/j.cell.2005.01.014 15766527

[14] Huarte M, Rinn JL, “Large non-coding RNAs missing links in cancer?” *Hum Mol Gener*, vol. 19, no. R2, pp.R152–R161, 2010. 10.1093/hmg/ddq353 PMC2953740 20729297

[15] Bhan A, Soleimani M, “Long Noncoding RNA and cancer: a new paradigm” Cancer Research, vol. 77, no. 15, pp. 3965–3981, 2017. doi: 10.1158/0008-5472.CAN-16-2634 28701486 PMC8330958

[16] Chevli KK, Duff M, et al., “Urinary PCA3 as a predictor of prostate cancer in a cohort of 3,073 men under- going initial prostate biopsy,” *J Urol*, vol. 191, no. 6, pp. 1743–1748, 2014. doi: 10.1016/j.juro.2013.12.005 24333241

[17] Salmena L., Polisene L., et al., “A ceRNA hypothesis: the Rosetta Stone of a hidden RNA language?” *Cell*, vol. 146, no. 3, pp. 353–358, 2011. doi: 10.1016/j.cell.2011.07.014 21802130 PMC3235919

[18] Zhang J., Fan D., Jian Z., et al., “Cancer specific long noncoding RNAs show differential expression patterns and competing endogenous RNA potential in hepatocellular carcinoma,” *PLoS One*, vol. 10, no. 10: e0141042, 2015. doi: 10.1371/journal.pone.0141042 26492393 PMC4619599

[19] Sui J., Li Y. H., Zhang Y. Q., et al., “Integrated analysis of long noncoding RNA associated ceRNA network reveals potential lncRNA biomarkers in human lung adenocarcinoma,” *Int J Oncol*, vol. 49, no. 5, pp. 2023–2036, 2016. doi: 10.3892/ijo.2016.3716 27826625

[20] Peng W. Z., Si S., Zhang Q. X., et al., “Long non-coding RNA MEG3 functions as a competing endogenous RNA to regulate gastric cancer progression” Journal of Experimental & Clinical Cancer Research, vol. 34, no. 1: 79, 2015. 10.1186/s13046-015-0197-7 PMC4529701 26253106

[21] Zhang Z. P., Xu Y. J., et. al., “Comprehensive characterization of lncRNA-mRNA related ceRNA network across 12 major cancers,” Oncotarget, vol. 7, no. 39, pp. 64148–64167, 2016. doi: 10.18632/oncotarget.11637 PMC5325432 27580177

[22] Ru P., Steel R., Newhall P., et al., “miRNA-29b suppresses prostate cancer metastasis by regulating epithelial-mesenchymal transition signaling,” Mol Cancer Ther, vol. 11, no. 5, pp. 1166–1173, 2012. doi: 10.1158/1535-7163.MCT-12-0100 22402125

[23] Musumeci M., Coppola V., Addario A., et al., “Control of tumor and microenvironment cross-talk by miR-15a and miR-16 in prostate cancer,” Oncogene. Vol. 30, no. 41, pp. 4231–4242, 2011. 10.1038/onc.2011.140 21532615

[24] Liu C., Kelnar K., Liu B., et al., “The microRNA miR-34a inhibits prostate cancer stem cells and metastasis by directly repressing CD44,” Nat Med, vol. 17, no. 2, pp. 211–215, 2011. doi: 10.1038/nm.2284 21240262 PMC3076220

[25] Zhang B. and Horvath S., “A general framework for weighted gene co-expression network analysis,” *Statistical Applications in Genetics and Molecular Biology*, vol. 4, no. 1: 17, 2005. 10.2202/1544-6115.1128 16646834

[26] Langfelder P., Luo R., Oldham M. C., Horvath S. (2011), “Is my network module preserved and reproducible?” *PloS Comp Biol*, vol. 7, no. 1: e1001057. 10.1371/journal.pcbi.1001057 PMC3024255 21283776

[27] Langfelder P. and Horvath S., “WGCNA: an R package for weighted correlation network analysis,” *BMC Bioinformatics*, vol. 9, no. 1: 559, 2008. 10.1186/1471-2105-9-559 PMC2631488 19114008

[28] Jeggari A., Marks D.S. and Larsson E., “miRcode: a map of putative microRNA target sites in the long non-coding transcriptome,” *Bioinformatics*, vol. 28, no. 15, pp. 2062–2063, 2012. doi: 10.1093/bioinformatics/bts344 22718787 PMC3400968

[29] Agarwal V., Bell G.W., Nam J.W. and Bartel D.P., “Predicting effective microRNA target sites in mammalian mRNAs,” *eLife*, vol. 4, 2015. 10.7554/eLife.05005 PMC4532895 26267216

[30] Wong N. and Wang X., “miRDB: an online resource for microRNA target prediction and functional annotations,” *Nucleic Acids Res*, vol.43, no.1, pp. 146–152, 2014. 10.1093/nar/gku1104 PMC4383922 25378301

[31] Yu G., Wang L. G., et al., “clusterProfiler: an R package for comparing biological themes among gene cluster,” *OMICS: A Journal of Integrative Biology*, vol. 16, no. 5, pp. 284–287, 2012. doi: 10.1089/omi.2011.0118 22455463 PMC3339379

[32] La Vecchia C. and Negri E., “THYROID CANCER The thyroid cancer epidemic-overdiagnosis or a real increase?” *NATURE REVIEWS ENDOCRINOLOGY*, vol. 13, no. 6, pp. 318–319, 2017. doi: 10.1038/nrendo.2017.53 28450748

[33] Mo B. Y., Guo X. H., Yang M. R., et al., “Long non-coding RNA GAPLINC promotes tumor-like biologic behaviors of fibroblast-like synoviocytes as microRNA sponging in rheumatoid arthritis patients,” *Frontiers in Immunology*, vol. 9, no. 702, 2018. 10.3389/fimmu.2018.00702 PMC5902673 29692777

[34] Wang Y., Zeng X. D., Wang N. N., et al., “Long noncoding RNA DANCR, working as a competitive endogenous RNA, promotes ROCK1-mediated proliferation and metastasis via decoying of miR-335-5p and miR-1972 in osteosarcoma,” *Molecular Cancer*, vol. 17, no. 89, 2018. 10.1186/s12943-018-0837-6 PMC5948795 29753317

[35] Matson D. R., Hardin H., Buehler D., et al., “AKT activity is elevated in aggressive thyroid neoplasma where it promotes proliferation and invasion,” *Exp Mol Pathol*, vol. 103, no. 3, pp. 288–293, 2017. doi: 10.1016/j.yexmp.2017.11.009 29169802

[36] Gou L. S., Zou H. W. and Li B. B., “Long noncoding RNA MALAT1 knockdown inhibits progression of anaplastic thyroid carcinoma by regulating miR-200a-3p/FOXA1,” *Cancer Biology & Therapy*, vol. 20, no. 11, pp. 1355–1365, 2019. 31500506 10.1080/15384047.2019.1617567 PMC6804806

[37] Lin S. J., Tan L. P., Luo D. Y., et al., “Linc01278 inhibits the development of papillary thyroid carcinoma by regulating miR-376c-3p/DNM3 axis,” *Cancer Management and Research*, vol. 11, pp. 8557–8569, 2019. doi: 10.2147/CMAR.S217886 31572010 PMC6756842

[38] Sui G. Q., Zhang B. T., et al., “The lncRNA SNHG3 accelerates papillary thyroid carcinoma progression via the miR-214-3p/PSMD10 axis,” *Journal of Cellular Physiology*, vol. 235, no. 10, pp. 6615–6624, 2020. doi: 10.1002/jcp.29557 32048306

[39] Li Y. Y., Chen M. J. and Liu C. P., “Metabolic changes associated with papillary thyroid carcinoma: A nuclear magnetic resonance-based metabolomics study,” *International Journal of Molecular Medicine*, vol. 41, no. 5, pp. 3006–3014, 2018. doi: 10.3892/ijmm.2018.3494 29484373

[40] Nozhat Z., Hedayati M., “PI3K/AKT Pathway and Its Mediators in Thyroid Carcinomas,” *Molecular Diagnosis & Therapy*, vol. 20, no. 1, pp. 13–26, 2016. 26597586 10.1007/s40291-015-0175-y

[41] Wang P., Gu J. L., Wang K. J., et al., “miR-206 inhibits thyroid cancer proliferation and invasion by targeting RAP1B,” *Journal of Cellular Biochemistry*, vol. 120, no. 11, pp. 18927–18936, 2019. doi: 10.1002/jcb.29213 31245877

[42] Zhang H.D., Jiang L. H., Sun D. W., et al., “MiR-139-5p: promising biomarker for cancer,” *Tumor Biology*, vol. 36, no. 3, pp. 1355–1365, 2015. doi: 10.1007/s13277-015-3199-3 25691250

[43] Zheng Y., Zheng B. W., Meng X., et al., “LncRNA DANCR promotes the proliferation, migration, and invasion of tongue squamous cell carcinoma cells through miR-135a-5p/KLF8 axis,” *Cancer Cell International*, vol.19, no.1: 302, 2019. 10.1186/s12935-019-1016-6 PMC6862788 31827393

[44] Yang H., Fang F., Chang R. M., et al., “MicroRNA-140-5p Suppresses Tumor Growth and Metastasis by Targeting Transforming Growth Factor beta Receptor 1 and Fibroblast Growth Factor 9 in Hepatocellular Carcinoma,” *Hepatology*, vol. 58, no. 1, pp. 205–217, 2013. doi: 10.1002/hep.26315 23401231

[45] Hsieh T. H., Hsu C. Y., Tsai C. F., et al., “miR-125a-5p is a prognostic biomarker that targets HDAC4 to suppress breast tumorigenesis,” *Oncotarget*, vol. 6, no.1, pp. 495–509, 2015. 10.18632/oncotarget.2674 PMC4381610 25504437

[46] Li W. Y., Hu S. and Tian C. F., “TRIP4 transcriptionally activates DDIT4 and subsequent mTOR signaling to promote glioma progression,” *Free Radical Biology and Medicine*, vol. 177, pp. 31–47, 2021. doi: 10.1016/j.freeradbiomed.2021.10.009 34648907

[47] Luo J. H., Zhu Y. H. and Xiang C., “Favorable function of Ectonucleoside triphosphate diphosphohydrolase 1 high expression in thyroid carcinoma,” *Hereditas*, vol. 158, no. 1: 33, 2021. 10.1186/s41065-021-00198-6 PMC8408975 34465393

[48] Gomez A., Colombo R., Pontoglio A., et al., “Functional analysis of six uncharacterised mutations in LDLR gene,” Atherosclerosis, vol. 291, pp. 44–51, 2019. doi: 10.1016/j.atherosclerosis.2019.10.013 31689621

[49] Obara Y., Imai T., Sato H., et al., “Midnolin is a novel regulator of parking expression and associated with Parkinson’s Disease,” Scientific reports, vol. 7, 5885, 2017. doi: 10.1038/s41598-017-05456-0 28724963 PMC5517452

[50] Chen X., Wang L., Qu J., et al., “Predicting miRNA-disease association based on inductive matrix completion,” *Bioinformatics*, vol. 34, no. 24, pp. 4256–4265, 2018. doi: 10.1093/bioinformatics/bty503 29939227

[51] Zhang L., Liu T., Chen H. Y., et al., “Predicting lncRNA-miRNA interactions based on interactome network and graphlet interaction,” *Genomics*, vol. 113, no. 3, pp. 874–880, 2021. doi: 10.1016/j.ygeno.2021.02.002 33588070

[52] Zhang L., Yang P. Y., Feng H. W., et al., “Using Network Distance Analysis to Predict lncRNA-miRNA Interactions,” *Interdiscip Sci*, vol. 13, no. 3,pp. 535–545, 2021. doi: 10.1007/s12539-021-00458-z 34232474

[53] Liu H. S., Ren G. F., Chen H. Y., et al., “Predicting lncRNA-miRNA interactions based on logistic matrix factorization with neighborhood regularized,” *Knowledge-based system*,vol. 191, 105261, 2019.

[54] Wang CC, Han CD, Zhao Q, Chen X. Circular RNAs and complex diseases: from experimental results to computational models. *Brief Bioinform*, vol. 22, no. 6, 2021. doi: 10.1093/bib/bbab286 34329377 PMC8575014

[55] Liu W, Jiang Y, Peng L, Sun X, Gan W, Zhao Q, et al. “Inferring Gene Regulatory Networks Using the Improved Markov Blanket Discovery Algorithm”. *Interdiscip Sci*, vol. 14, no. 1, pp. 168–181, 2022. doi: 10.1007/s12539-021-00478-9 34495484

[56] Sauerbrei Willi, Taube Sheila E, McShane Lisa M, Cavenagh Margaret M, Altman Douglas G, “Reporting Recommendations for Tumor Marker Prognostic Studies (REMARK): An Abridged Explanation and Elaboration,” JNCI: *Journal of the National Cancer Institute*, vol. 110, no.8, pp. 803–811, 2018. doi: 10.1093/jnci/djy088 29873743 PMC6093349

